# Resident burnout: evaluating the role of the learning environment

**DOI:** 10.1186/s12909-018-1166-6

**Published:** 2018-03-27

**Authors:** Stefan N. van Vendeloo, Lode Godderis, Paul L. P. Brand, Kees C. P. M. Verheyen, Suria A. Rowell, Harm Hoekstra

**Affiliations:** 1 Department of Orthopedic surgery and Traumatology, Isala Hospital, Dokter van Heesweg 2, Zwolle, NL-8025 AB the Netherlands; 2 Department of Public Health and Primary Care, Environment and Health, Leuven, Belgium; 3 IDEWE, external service for prevention and protection at work, Heverlee, Belgium; 4 Princess Amalia Children’s Centre, Isala Hospital, Zwolle, The Netherlands; 5 UMCG Postgraduate School of Medicine, University Medical Centre, Hanzeplein 1, 9713 GZ Groningen, the Netherlands; 6 Department of Development and Regeneration, KU Leuven - University of Leuven, Leuven, Belgium

**Keywords:** Resident burnout, Resident well-being, Quality of life, Learning environment, Competency-based education

## Abstract

**Background:**

Although burnout is viewed as a syndrome rooted in the working environment and organizational culture, the role of the learning environment in the development of resident burnout remains unclear. We aimed to evaluate the association between burnout and the learning environment in a cohort of Belgian residents.

**Methods:**

We conducted a cross-sectional online survey among residents in a large university hospital in Belgium. We used the Dutch version of the Maslach Burnout Inventory (UBOS-C) to assess burnout and the Dutch Residency Educational Climate Test (D-RECT) to assess the learning environment.

**Results:**

A total of 236 residents (29 specialties) completed the survey (response rate 34.6%), of which 98 (41.5%) met standard criteria for burnout. After multivariate regression analysis adjusting for hours worked per week, quality of life and satisfaction with work-life balance, we found an inverse association between D-RECT scores and the risk of burnout (adjusted odds ratio; 0.47 for each point increase in D-RECT score; 95% CI, 0.23 – 0.95; *p* = 0.01).

**Conclusions:**

Resident burnout is highly prevalent in our cohort of Belgian residents. Our results suggest that the learning environment plays an important role in reducing the risk of burnout among residents.

## Background

Burnout is a work-related syndrome that is primarily driven by workplace stressors [1]. Three dimensions define burnout: emotional exhaustion, depersonalization and a diminished sense of personal accomplishment [2]. Although individual traits might play a role in the development of physician burnout, a recent meta-analysis indicates that organization-directed approaches are more effective in reducing burnout compared to individual interventions [3]. This finding supports the hypothesis that burnout is rooted in issues related to working environment and organizational culture, instead of being an individual problem.

Burnout among medical residents is highly prevalent [4–6]. High educational demands, long working hours, lack of autonomy, a high level of work-home interference, a lack of reciprocity in professional relationships and uncertainty about the future are common explanations [7–10]. Rates between 25 and 60% have been reported in a wide spectrum of medical specialties [11]. These large ranges can be attributed to the use of different definitions, measurements and study designs [12]. Resident burnout is a major concern because it has serious consequences on patient outcomes and on the personal lives of residents. Importantly, burnout is linked with an increase in medical errors and reduced quality of patient care [7, 13, 14]. Furthermore, residents who suffer from burnout have an increased risk of substance abuse and suicidal ideation [15].

Burnout could be an obvious outcome of the context residents participate in during their day-to-day work as a doctor [16]. This context is also known as the learning environment, which is a complex construct that includes formal and informal aspects of the training program, as well as the overall atmosphere [17] and organizational aspects within the teaching hospital [18]. The way people in a particular department approach the process of learning is considered a reflection of the learning environment [19]. This learning environment is thought to play a key role in the development of residents towards independent practice [20]. It has been hypothesized that the learning environment could be an important driver of burnout [6, 17, 20, 21]. Although specific characteristics of the learning environment appear to be critically influencing medical student burnout [22], the role the learning environment plays in resident burnout is less clear. Furthermore it is unknown which characteristics of the learning environment are associated with symptoms of burnout in residents.

We aimed to assess how the perceived quality of the learning environment relates to burnout in a sample of Belgian residents from different specialties in a single academic hospital. Secondarily we set out to determine which characteristics of the learning environment are associated with resident burnout. Moreover, we aimed to determine the effect size of the learning environment by controlling for several demographic and occupational predictors of resident burnout. We hypothesized that a consistent and relevant association exists between the perceived quality of the learning environment and burnout.

## Methods

All residents that were enrolled in one of the postgraduate medical training programs in the University Hospitals Leuven in Belgium on the 1st of December 2016 received an invitation by email to participate in the study and complete an online self-report survey. Residents were recruited from this single academic center because all specialty training programs share the same educational framework. The invitation was sent by the local association of residents to guarantee anonymity. Residents received a reminder after 2 weeks if the survey was not completed by that time. The local ethical board determined that the study was exempt from formal ethical review. This study was carried out according to the ethical principles for medical research involving human subjects of the WMA Declaration of Helsinki. No individual data were collected, anonymity was guaranteed, participation was voluntary, and informed consent was obtained.

### Learning environment

To assess the quality of the learning environment we used the recently revised version of the Dutch Residency Educational Climate Test (D-RECT) [23]. The revised D-RECT is a validated 35-itemd questionnaire to assess 9 subscales of the learning environment. The 9 subscales that comprise the D-RECT are: educational atmosphere, teamwork, role of specialty tutor, coaching and assessment, formal education, resident peer collaboration, work is adapted to resident’s competence, accessibility of supervisors and patient sign-out. Respondents are asked to indicate their agreement on a scale ranging from 1 (totally disagree) to 5 (totally agree).

### Burnout

We used the validated Dutch version (UBOS-C) [24] of the Maslach Burnout Inventory (MBI) to assess burnout. This instrument consists of 20 items covering the three dimensions of burnout: emotional exhaustion (8 items), depersonalization (5 items) and personal accomplishment (7 items). Items were scored on a 7-point Likert scale ranging from ‘never’ (0) to ‘always’ (6). Mean scores were calculated for each dimension. Cut-off scores were used for ascertainment of burnout, based on a reference group of 10,552 Dutch healthcare employees [23]. A resident was diagnosed with burnout if there was either a mean score ≥ 2.50 on emotional exhaustion and ≥ 1.80 (men) or ≥ 1.60 (women) on depersonalization, or a mean score ≥ 2.50 on emotional exhaustion and a mean score of ≤ 3.70 on personal accomplishment [25].

### Demographic and training-related characteristics

Respondents provided information on: gender, age, type of medical specialty, year of postgraduate training, total number of hours spent working per week and the number of hours per week spent on clinical (patient related) activities, administrative (non-clinical) activities and activities related to training. Furthermore, we measured residents’ overall quality of life using a single-item linear analogue self-assessment (scale 1 to 5, with response options ranging from “As bad as it can be” to “As good as it can be”) and residents’ satisfaction with their work-life balance using a similar 5-point scale. These instruments are validated and widely used in quality of life research [2].

### Statistical analysis

All analyses were done using SPSS version 17 (SPSS Inc., Chicago, Illinois, US). Standard descriptive summary statistics were used to characterize the sample. Evaluations that were missing more than 17 items (> 50% of total items) were excluded from further analysis. The remaining missing values were assumed to be missing at random and imputed by expectation maximization. Independent Student’s t tests (continuous variables) were computed to compare means. Multivariate logistic regression analysis was conducted to evaluate the association between learning environment (D-RECT scores) and resident burnout, adjusted for potential predictors of burnout. Predictors of burnout were determined in a univariate analysis. In the multivariate model, we adjusted factors that were independently associated with burnout. None of the included variables were associated with the D-RECT scores, thus the assumption of linearity was not violated. All tests used were two-tailed and *p*-values < 0.05 were considered significant.

## Results

### Sample descriptive statistics

Of the 682 residents who received an invitation, 252 participated. A total of 16 evaluations were excluded due to missing values, which gave us a sample of 236 (29 different specialties) evaluations (response rate 34.6%). A total of 98 (41.5%) residents fulfilled the standard criteria for burnout. One hundred twenty five residents (53.0%) scored high on the scale of emotional exhaustion, 125 (53.0%) scored high on the scale of depersonalization, and 60 (23.4%) scored low on the scale of personal accomplishment. The mean score (SD) of the D-RECT was 2.65 (0.43). The median age of the residents was 28 years (range 26 – 40). Residents spent an average of 60.9 (SD; 10.1) hours working per week. A total of 32.6 (SD; 14.9) hours per week were spent on clinical activities, 23.4 (SD; 14.2) on administrative activities and 4.3 (SD; 5.5) on training related activities. Residents with burnout spent an average of 2.61 working hours more per week compared to those without burnout (95% CI of difference; − 5.23 to 0.004, *p* = 0.05). We found no association between burnout and the total number of hours per week spent on clinical (patient related) activities (*p* = 0.60), training activities (*p* = 0.08) or non-training activities (e.g. administrative tasks) (*p* = 0.25). Table 1 shows the demographic and occupational characteristics of the responding residents and their association with burnout.

**Table 1:** Demographic and occupational characteristics of participating residents and associations (Pearson’s chi squared tests) between demographic, occupational and quality of life characteristics and burnout

	Total number of participating residents (% of total)	Number of residents with burnout (% of total)	*p*-value of difference in burnout rate per characteristic
Gender			
Male	96 (40.7)	45 (46.9)	
Female	140 (59,3)	53 (37.9)	
			0.17
Years in training			
1	52 (22.2)	22 (42.3)	
2	23 (9.7)	11 (47.8)	
3	31 (13.1)	8 (25.8)	
4	50 (21.2)	24 (48.0)	
5	45 (19.1)	18 (40.0)	
6	25 (10.6)	11 (44.0)	
7	8 (3.4)	2 (25.0)	
			0.49
Type of specialty			
Surgical	57 (24.2)	26 (45.6)	
Medical	144 (61.0)	59 (41.0)	
Supportive	34 (14.4)	12 (35.3)	
missing	1 (0.4)		
			0.62
Satisfaction with work/life balance			
Very dissatisfied	31 (13.1)	21 (67.7)	
Dissatisfied	4 (1.7)	2 (50.0)	
Neutral	69 (29.2)	14 (20.3)	
Satisfied	130 (55.1)	61 (46.9)	
Very satisfied	2 (0.8)	0 (0.0)	
			< 0.001
Quality of life			
As bad as it can be	12 (5.1)	10 (83.3)	
Bad	59 (25.0)	37 (62.7)	
Neutral	79 (33.5)	37 (46.8)	
Good	77 (32.6)	14 (18.2)	
As good as it can be	9 (3.8)	0 (0.0)	
			< 0.001
Burned out^a^	98 (41.5)		
Emotionally exhausted^b^	125 (53.0)		
Depersonalized^b^	125 (53.0)		
Reduced personal accomplishment^b^	60 (23.4)		

^a^Represents a high score (>75th percentile of reference group, Schaufeli ea.) on emotional exhaustion, combined with a high score on depersonalization and/or a low score (<25th percentile of reference group) on personal accomplishment

^b^Cut-off scores are determined as >75th percentile of the reference group (Schaufeli ea.) for emotional exhaustion and depersonalization and < 25th percentile of the reference group for reduced personal accomplishment

### Multivariate analysis

We used univariate analyses to identify demographic and occupational predictors of burnout. We found that gender (*p* = 0.17), year of training (*p* = 0.49), type of specialty (*p* = 0.62) and age (*p* = 0.32) were not associated with burnout. However, we found that the true number of hours worked per week (*p* = 0.05), satisfaction with work-life balance (*p* < 0.001) and overall quality of life (*p* < 0.001) were associated with burnout. After controlling for these predictors of resident burnout in a multivariate regression analysis, we found an inverse relationship between the mean D-RECT score and the risk of burnout (adjusted odds ratio, 0.47 for 1-point increase in D-RECT score; 95% CI, 0.23 – 0.95; *p* = 0.01).

### Bivariate analysis

We found that residents without burnout gave significantly higher D-RECT scores (mean, SD; 2.71, 0.39) than residents with burnout (mean, SD; 2.56, 0.46) (95% confidence interval for difference; 0.03 to 0.46, *p* = 0.006). The difference in D-RECT score between residents with and without burnout can be explained by a difference in D-RECT score for the dimension of emotional exhaustion and depersonalization (Table 2).

**Table 2:** Association between the 3 dimensions of burnout and overall burnout and the mean overall D-RECT scores (learning environment) in Belgian residents

		Mean score D-RECT (SD)	95% CI of difference	p-value
Emotional exhaustion	Exhausted	2.57 (0.39)	0.06 to 0.27	0.003
	Not exhausted	2.74 (0.44)		
Depersonalization	Depersonalization	2.57 (0.41)	0.06 to 0.27	0.003
	No depersonalization	2.73 (0.42)		
Reduced personal accomplishment	Not competent	2.64 (0.36)	− 0.12 to 0.14	0.86
	Competent	2.65 (0.45)		
Overall burnout	Burnout	2.56 (0.46)	0.03 to 0.46	0.006
	No burnout	2.71 (0.39)		

### Subscales of the D-RECT

Regarding the subscales of the D-RECT, we found that the scores on the subscales ‘role of the specialty tutor’ and ‘coaching and assessment’ were significantly higher in residents without burnout compared to those who suffer from burnout (Table 3).

**Table 3:** Comparison (Student’s t-tests) of overall D-RECT scores (learning environment) and D-RECT subscale scores in residents with and without burnout. To adjust for multiple comparisons *p* values < 0.01 were considered significant

Subscale of the D-RECT	Mean score D-RECT in residents with burnout	Mean score D-RECT in residents without burnout	95% CI for difference	*p* value
Educational atmosphere	2.31	2.46	−0.04 – 0.33	0.12
Teamwork	2.80	3.02	−0,02 – 0.45	0.07
Role of specialty tutor	2.35^a^	2.59^a^	0.08 – 0.39	0.003^a^
Coaching and assessment	2.06^a^	2.53^a^	0.26 – 0.66	< 0.001^a^
Formal education	2.78	2.99	0,03 – 0.39	0.02
Resident peer collaboration	2.78	2.72	−0.35 – 0.22	0.64
Work is adapted to resident’s competence	3.00	3.06	−0.16 – 0.27	0.61
Accessibility of supervisors	2.95	2.66	−0.54 – − 0.02	0.03
Patient sign-out	2.86	2.85	−0.26 – 0.25	0.91
Overall D-RECT score	2.56^a^	2.71^a^	0.05 – 0.26	0.006^a^

^a^Statistically significant

## Discussion

### Main findings

In this study, we examined the association between the perceived quality of the learning environment and resident burnout. We found that, even after adjusting for predictors of burnout, there was a significant and relevant association between the learning environment and burnout in our sample of Belgian residents from 29 different specialties.

### Environmental factors associated with burnout

Our multivariate analysis suggests that there is a significant and strong exposure-response relationship (OR 0.47) between the learning environment and burnout. Analysis of the subscales that comprise the D-RECT (learning environment) shows environmental factors that explain the association between learning environment and burnout are the subscales ‘role of the specialty tutor’ and ‘coaching and assessment’. The environmental factor role of the specialty tutor focuses on the behaviour of the supervisor. The amount of support from faculty members and the perception of being mistreated are strongly related to burnout among medical students [22]. Stressful relationships with supervisors [5] and insufficient autonomy [26] are in a similar way associated with burnout in residents. In contrast, residents who find their relationship with their supervisors mutually supportive and beneficial have fewer symptoms of burnout than trainees who feel under-appreciated by their supervisors [27]. Our results regarding the role of supervisory support in resident burnout are in agreement with these findings. The environmental factor ‘coaching and assessment’ is concerned with feedback, assessment of medical and general competencies and with supervisors evaluating whether a resident’s performance in patient care is in line with that resident’s level of training. Our results underline previous reports on the importance of regular feedback in the prevention of burnout [28]. When regular feedback regarding preparedness is lacking, residents might feel uncertain about whether they are prepared to perform a specific task [29], which could drive burnout.

### Learning environment scores explained

A recent study found a considerably higher rating for the quality of the learning environment in a large cohort of Dutch residents compared to the rating we found in our study [30]. We were able to use the same instrument (D-RECT) to assess the learning environment because the residents participating in our study share the same language as their Dutch counterparts. We have to acknowledge that the D-RECT has not been validated in the Belgian context and minor language and culture differences might affect the interpretation of some of the items. Nevertheless, we think that these minor differences cannot explain the large difference in D-RECT scores between the two studies. Little is known about what a specific score in the D-RECT says about the underlying construct, but score differences between teaching hospitals could guide initiatives to improve the learning environment. Several educational differences exist that could partly explain this difference in the perceived quality of the learning environment between Belgian and Dutch residents. Educational programs in the Netherlands have been modernized to become competency-based [31]. These competency-based programs promote learner-centeredness and shift the focus away from what is taught, towards what is learnt. The Belgian curriculum is mainly time-based and, although initiatives have been taken to put more emphasis on the development of generic competencies, appraisal tools have yet to be introduced. Novel coaching and assessment tools, that have been introduced and are broadly used in the Netherlands [31], generate more attention to feedback, which is likely appreciated by residents and reflected in higher D-RECT scores. On the other hand, we have to note that based on our study results, we are unable to determine which environmental factors are responsible for a difference in D-RECT score between the Belgian and Dutch residents. Moreover, the learning environment is believed to form the center of educational change [17] and the effectiveness of competency-based innovations seems to benefit from a supportive learning environment [20]. Hence, more research is needed to understand the dynamics of the relationship between educational innovations and perceived quality of the learning environment.

### Burnout prevalence

We found a burnout rate of 41%, which is much higher than the rate of 21% that was found in a national Dutch study [1], which used the same instrument and criteria for burnout as we did. This can be explained by the shorter workweek of 48 h of Dutch residents, which is reasonably lower than the workweek we found our cohort: 60.9 h per week. We have to state that the difference in work hours between residents with burnout and without burnout was relatively small in the current study (2.6 h), which makes it doubtful whether this difference is relevant. The effect of number of hours worked on the development of resident burnout remains controversial, but a recent study indicates that a higher number of work hours does increase the risk of resident burnout [7]. An earlier study conducted in Belgium, including residents from multiple teaching hospitals and using the same instrument to assess burnout (UBOS-C), found a rate of 33.5% resident burnout [3], which is still lower than the rate we found. This difference in burnout rate can be explained by the fact that our study was conducted in a single academic center, instead of including multiple hospitals.

### Practical implications and future research

The results of a recent meta-analysis suggest that burnout among physicians, including residents, is driven by organizational factors rather than individual factors [15]. These results are in agreement with insights that burnout is not an indication of personal failing but rather of a failing working and social environment [32]. This environment includes aspects of the workload, schedule, communication, workflow and teamwork [15, 33]. Several training-related factors, like high educational demands and lack of autonomy, pose an additional risk to residents when compared to physicians that are not in training [1]. Moreover, some even state that burnout is the obvious outcome of the disconnect between medical training programs and the realities of the need to work with colleagues, hospital personnel and patients who have different visions of how the healthcare organization should operate [16]. We have to note however, that burnout is assumed to be the result of a chronic imbalance between job demands and job resources [13]. Hence, we cannot conclude that resident burnout is simply caused by a poor learning environment. Nevertheless, we do believe that the learning environment plays an important role in the motivational process of residents, because a healthy learning environment fosters growth, learning and development [17, 20, 22]. Initiatives to improve the learning environment and contribute to burnout prevention should preferably address the learning environment as a whole and focus in particular on improving supervisory support and improve the quality of coaching and assessment. This could be achieved by the implementation of faculty staff development, which provides faculty members with requisite pedagogic tools needed to enhance supervisory performance (e.g. giving feedback, application of coaching and assessment), establish an optimal learning environment and enable them to detect and respond to emotional distress [34]. Additional research is needed to explore causal relationships between the environmental factors associated with burnout and to determine which approach has the highest potential for minimising resident burnout. Future prospective studies could be designed that randomise residents to a training program that pays attention to more generic competencies, versus a more traditional program, because training generic competencies has shown to reduce the risk of burnout in junior medical specialists [35]. These study designs could also evaluate new assessment and appraisal tools, because they are thought to aid in better preparing residents for practice and improve their well-being in the end [20, 35].

### Strengths and limitations

The current study is the first to describe a consistent association between the learning environment and burnout among residents using the complete MBI and D-RECT rather than abbreviated versions. We were able to adjust for work hours, quality of life and work life balance, which allowed a robust analysis of the quality of the learning environment and the development of burnout. This study has several limitations. The response rate of 34.5% was relatively low, although it is higher than most other survey studies using online questionnaires [1, 4]. The absolute difference in D-RECT (learning environment) scores we found when comparing residents with and without burnout was rather small. It could be possible that this difference, although significant, is too small to be relevant. What argues against this explanation is the fact that the D-RECT makes use of a 5-point Likert scale, which restricts residents in their ability to indicate improvement as they only have very few options to discriminate between levels of agreement with every item. Earlier work on learning environment using the same instrument has shown similar significant but small differences [18]. Our cross-sectional study design precludes determination of whether the learning environment is causally related to burnout. It could be possible that residents who suffer from burnout are simply more likely to give a lower rating for the learning environment. However, only specific aspects of the learning environment (role of the specialty tutor, coaching and assessment) were associated with burnout and other aspects were not, which argues against an overall lower rating of the learning environment by residents with burnout. Furthermore, we didn’t include an instrument to evaluate personality traits, which is thought to be a risk factor of resident burnout. Nevertheless, the extent to which personality relates to the development of resident burnout has yet to be determined and organizational factors are thought to play a more important role than individual factors [15]. Moreover, our study was conducted at a single institution, limiting the generalizability of our results. Finally, despite the high response rate our results are possibly limited by response bias. Residents with burnout could be more likely to complete questionnaires regarding burnout because the topic is relevant to them, but they could also be to apathetic to complete the questionnaire. The way resident burnout influences response rate is unknown.

## Conclusions

We found a consistent association between the perceived quality of the learning environment and burnout in residents. Given the serious personal and professional ramifications of resident burnout, there is a need for interventions addressing factors of the learning environment that drive burnout. It is desirable that these include improvement of supervisory support and coaching and assessment. Future research should evaluate which organization-directed approaches are most effective in preventing resident burnout.

## Abbreviations

D-RECT: Dutch residency educational climate test
MBI: Maslach burnout inventory
UBOS-C: Utrechtse burnout schaal voor contactuele beroepen
